# Xenogeneic-Free System for Biomanufacturing of Cardiomyocyte Progeny From Human Pluripotent Stem Cells

**DOI:** 10.3389/fbioe.2020.571425

**Published:** 2020-10-23

**Authors:** Preeti Ashok, Abhirath Parikh, Chuang Du, Emmanuel S. Tzanakakis

**Affiliations:** ^1^Chemical and Biological Engineering, Tufts University, Medford, MA, United States; ^2^Kite Pharma, Gilead, Santa Monica, CA, United States; ^3^Biomedical Engineering, Tufts University, Medford, MA, United States; ^4^Clinical and Translational Science Institute, Tufts Medical Center, Boston, MA, United States; ^5^Developmental Molecular and Chemical Biology, Graduate School of Biomedical Sciences, Tufts University School of Medicine, Boston, MA, United States

**Keywords:** human pluripotent stem cells, cardiomyocytes, biomanufacturing, xeno-free culture, bioreactor

## Abstract

Functional heart cells and tissues sourced from human pluripotent stem cells (hPSCs) have great potential for substantially advancing treatments of cardiovascular maladies. Realization of this potential will require the development of cost-effective and tunable bioprocesses for manufacturing hPSC-based cell therapeutics. Here, we report the development of a xeno-free platform for guiding the cardiogenic commitment of hPSCs. The system is based on a fully defined, open-source formulation without complex supplements, which have varied and often undetermined effects on stem cell physiology. The formulation was used to systematically investigate factors inducing the efficient commitment to cardiac mesoderm of three hPSC lines. Contractile clusters of cells appeared within a week of differentiation in planar cultures and by day 13 over 80% of the cells expressed cardiac progeny markers such as TNNT2. In conjunction with expansion, this differentiation strategy was employed in stirred-suspension cultures of hPSCs. Scalable differentiation resulted in 0.4–2 million CMs/ml or ∼5–20 TNNT2-positive cells per seeded hPSC without further enrichment. Our findings will contribute to the engineering of bioprocesses advancing the manufacturing of stem cell-based therapeutics for heart diseases.

## Introduction

Heart disease, which is a major cause of morbidity and mortality (Benjamin et al., 2018), is associated with loss of cardiac myocytes and declining function eventually leading to organ failure. Cardiomyocytes (CMs) exhibit scant, if any, regenerative capacity and the existence of adult heart-residing stem cells capable of repairing cardiac injury has been called into question (van Berlo et al., 2014). Moreover, the shortage of donor organs and the required life-long immunosuppression are limiting factors for heart transplantation. To this end, human pluripotent stem cell (hPSC)-based cellular therapies hold promise for reconstituting the damaged cardiac tissue and improving its function. The need for >10^9^ billion cells to repair the myocardium (Addis and Epstein, 2013) after infarction calls for the development of scalable methods for the efficient conversion of hPSCs to functional CMs.

Drawing on knowledge of embryonic heart development (Ueno et al., 2007; Parikh et al., 2015), cardiogenic differentiation of cultured hPSCs involves their transition through mesendoderm, cardiac mesoderm, and eventually cardiac cell fates. The sequential activation and inhibition of canonical Wnt/β-catenin signaling has reliably led to the derivation of CM-like cells from several hPSC lines. Various protocols differing in the type, concentration, and timing of addition or removal of differentiation factors have been developed to improve the efficiency of hPSC-to-CM conversion in conventional planar cultures (e.g., dishes). Many utilize non-physiological components [e.g., polyvinyl alcohol (Burridge et al., 2014)], chemically defined lipids with animal-sourced components (e.g., cholesterol), and complex supplements such as B27 (Lian et al., 2012). The latter, which was originally developed for neuronal cell cultivation (Brewer et al., 1993), contains an assortment of components including antioxidants, lipids, steroids, and bovine serum albumin (BSA), with potentially multifarious but largely unexplored roles in the specification of hPSCs to heart cells.

While such protocols have been applied directly to stirred-suspension cultivation (Kempf et al., 2014; Chen et al., 2015) despite marked differences with static culture modalities, the complex formulations involved make challenging to evaluate the impact of individual raw materials on the critical quality attributes of the hPSC-derived CMs and on indicators of performance of envisioned bioprocesses for cell therapy manufacturing. Use of such media also hinders tackling the inherent hPSC line-to-line variability and developing relevant customized solutions. Additionally, the substantial decline in cell viability – particularly at the onset of differentiation – can contribute to manufacturing-related impurities impacting the product quality and process outcome.

Here, a xeno-free culture system is reported for generating cardiac cell progeny in static and stirred-suspension cultures with combined hPSC propagation and differentiation. For this purpose, a fully defined medium without animal-derived inputs was developed that does not interfere with the acquisition of a mesodermal cardiac cell fate in the presence of physiologically relevant stimuli. This formulation allowed the systematic investigation of factors inducing the efficient differentiation to cardiac mesoderm of hPSCs arriving at a xeno-free regimen for cardiogenic commitment. In conjunction with expansion, this strategy was employed to guide the specification of three hPSC lines to heart cell progeny in a stirred-suspension cultivation process. Cells transitioned through developmentally relevant stages expressing characteristic markers and eventually yielding populations with over 85% CM-like cells without further lineage purification after less than 2 weeks. The findings will contribute to the engineering of scalable bioprocesses advancing the manufacturing of cell therapeutics for heart diseases.

## Materials and Methods

### Human Pluripotent Stem Cell Culture

The human induced-pluripotent stem cell (hiPSC) lines iPSC(IMR90)-4 (henceforth IMR90-4, WiCell, Madison, WI) and B12-3 (Harvard Stem Cell Institute, Cambridge, MA) and the embryonic stem cell (hESC) line H9 (WiCell) were maintained with xeno-free StemMACS medium (Miltenyi, Cambridge, MA) either on xeno-free vitronectin (VTN-N) (Invitrogen, Carlsbad, CA), or on Matrigel. Cells were manually passaged every 5–7 days at 1:4–1:6 ratios and media were replaced daily. The cultures were maintained in 5% CO_2_/95% air at 37°C. Cells were counted with a hemocytometer or the TC20 automated cell counter (Bio-Rad, Hercules, CA) and viability was determined via Trypan Blue dye exclusion (Life Technologies, Woburn, MA).

### Spinner Flask Culture of hPSCs

Human PSCs were cultured as aggregates in 250 ml spinner flasks (Corning Inc., Corning, NY) in 5% CO_2_/95% air at 37°C and 50 rpm. Cells were pretreated with 10 μM Rho-associated protein kinase (ROCK) inhibitor (Y-27632) (Enzo Life Sciences, Farmingdale, NY) for 1 h, dispersed using Accutase (Innovative Cell Technologies, San Diego, CA) and seeded at 2 × 10^5^ cells/ml in StemMACS medium with 10 μM ROCK inhibitor and 0.2% Pluronic F68 (Ashok et al., 2016). Two thirds of medium were replaced after 2 days, and then daily until day 5. Cells were passaged by dissociating aggregates to single cells as described above. IMR90-4 and B12-3 hiPSCs were seeded with 20 μL of animal origin-free coating Matrix (cat. no. R011K; Thermo Fisher Scientific, Waltham, MA) to promote clustering.

### Cardiomyocyte Differentiation of hPSCs

In preparation for differentiation cells were seeded at 4–8 × 10^5^ cells/cm^2^ and after 6 days of expansion, differentiation was induced (day 0) by washing the cells with PBS (Sigma-Aldrich, St. Louis, MO) and adding xeno-free differentiation medium (XF; Table 1) containing BMP4 and WNT3A (R&D Systems, Minneapolis, MN) at stated concentrations. The next day, this medium was replaced with XF medium without BMP4 and WNT3A. From day 2 to 8, cells were incubated in XF with 10 μM KY02111 (R&D Systems, Minneapolis, MN) replenished daily. From day 9 on cells were cultured in XF, which was replaced daily.

**TABLE 1:** Xeno-free medium (XF) composition.

Raw materials (all xeno-free)	Concentration	Purpose
DMEM basal medium (4.5 g/L) (Thermo Fisher Scientific, cat. no. 10569010)	100%	Basal medium containing salts, amino acids, vitamins, glucose
Recombinant human holo-transferrin (Millipore-Sigma, cat. no. T4132)	5 μg/ml	Iron carrier
Sodium Selenite (Millipore-Sigma, cat. no. S5261)	5 ng/ml	Increases glutathione peroxidase activity protecting from oxidative damage
Recombinant human serum albumin (rHA; Millipore-Sigma, cat. no. A9731)	0.05%	Carrier for nutrients
Sodium pyruvate (Thermo Fisher Scientific, cat. no. 11360070)	1 mM	Additional energy source
MEM non-essential amino acids (Thermo Fisher Scientific, cat. no. 11140050)	1×	Supports cell growth and proliferation
RPMI 1640 amino acid supplement (Millipore-Sigma, cat. no. R7131)	1×	Nutrients for cell mass maintenance
RPMI 1640 vitamin supplement (Millipore-Sigma, cat. no. R7256)	1×	Added to maintain cell metabolism and function

Differentiation in spinner flasks was carried out after 5 days of hPSC expansion as aggregates. Subsequently aggregates were transferred to centrifuge tubes and spun down at 100 × *g* for 5 min. The medium was aspirated, aggregates were washed with PBS, and XF was added containing 30 ng/ml BMP4 and 200 ng/ml WNT3A (alternatively, 6 μM CHIR99021 was added) before returning the aggregate suspension to the spinner flasks. Differentiation in the spinner flasks proceeded using the same media and timing as in dish cultures.

### RNA Extraction, RT-PCR and Quantitative PCR Analysis

Total RNA was extracted using Trizol (Life Technologies) according to manufacturer’s instructions. Reverse transcription was performed at 42°C for 60 min with 1 μg total RNA using ImProm-II reverse transcriptase (Promega, Madison, WI) and 250 ng oligo(dT)_12__–__18_ primers (Thermo Fisher Scientific, Waltham, MA). The resulting complimentary DNA was analyzed on a StepOne Plus qPCR thermocycler (Applied Biosystems, Foster City, CA) by quantitative PCR (qPCR) for 40 cycles and 58–60°C annealing temperature depending on primer set. Primer sequences are listed in Supplementary Table 1. Gene expression was analyzed with the ΔΔC_*T*_ method (Fan et al., 2014). *ACTB* served as the endogenous gene expression control.

### Immunocytochemistry

Cells were fixed in 4% paraformaldehyde (Millipore-Sigma, St. Louis, MO) for 20 min and permeabilized with 0.1% Triton X-100 in PBS for 20 min at room temperature for nuclear markers. Cell fixation and permeabilization for cytoskeletal markers was done with cold (−20°C) methanol for 3–5 min. Samples were washed three times (5 min each time) with PBS between each step and blocked with 3% normal donkey serum (NDS; Jackson ImmunoResearch Laboratories, West Grove, PA) in PBS for 30 min. Samples were incubated at 4°C with primary antibodies for: cardiac troponin T (TNNT2; rabbit; ab45932; Abcam, Cambridge, MA), α-actinin (ACTN1; mouse; sc-15335) and GATA4 (rabbit; sc-9053; both from Santa Cruz Biotechnology, Dallas, TX). Incubation with secondary antibodies was performed at room temperature for 1 h with donkey anti-rabbit or anti-mouse antibodies conjugated to DyLight 488 or 549 (Jackson ImmunoResearch Inc., West Grove, PA). Nuclear DNA was stained with DAPI (Sigma-Aldrich, St. Louis, MO). Controls were stained with IgG instead of with a primary antibody. Immunostaining was visualized with a Leica TCS SPE confocal microscope (Leica Microsystems Inc., Buffalo Grove, IL).

### Flow Cytometry

Cells were fixed for 10 min in 4% formaldehyde in PBS, washed three times with PBS, permeabilized with cytonin (Trevigen, Gaithersburg, MD) for 30 min and blocked with 3% NDS in PBS for another 30 min. Then, 1% NDS solution was added for 1 h at room temperature with primary antibodies: rabbit anti-cardiac TNNT2, rabbit anti-NKX2.5 (ab91196, Abcam) or murine anti-myosin heavy chain (MHC) MYH1E (MF20; Developmental Studies Hybridoma Bank, Iowa City, IA). After washing with 1% NDS three times, the samples were incubated with a donkey anti-rabbit secondary antibody conjugated to DyLight 488 (Jackson ImmunoResearch) for 1 h at room temperature in 1% NDS solution. Samples were analyzed with the Attune NxT flow cytometer (Thermo Fisher Scientific) and the FCS Express software (v. 7.0, *De Novo* Software, Glendale, CA). Gating was based on samples treated with isotype antibodies (control).

### Western Blot Analysis

Total protein was isolated using lysis buffer containing Tris–HCl (50 mM, pH 8), NaCl (150 mM), NP40 (1%), SDS (0.1%), sodium deoxycholate (1%), protease inhibitor cocktail including PMSF (Sigma-Aldrich), and phosphatase inhibitors (1 mM sodium fluoride, 5 mM sodium pyrophosphate, 5 mM sodium orthovanadate). Protein concentration was determined with the Bradford method (Pierce Biotechnology). Total protein (20 μg/lane) was loaded in 12% (w/w) SDS-PAGE along with a biotinylated protein ladder (Cell Signaling Technology, Beverly, MA). After protein transfer, the polyvinylidene fluoride (PVDF) membranes were blocked with blocking buffer consisting of 5% skim milk in TBS with 0.1% Tween (TBST). Rabbit GATA4 (sc-9053; Santa Cruz Biotechnology) or beta actin (ACTB; 4967; Cell Signaling Technology) antibody in blocking buffer was then added to samples for overnight incubation at 4°C. After four 10-min washes with TBST, the membranes were incubated for 1 h at room temperature with horseradish peroxidase (HRP)-linked donkey anti-rabbit and anti-biotin secondary antibodies and washed four times with TBST. Enhanced chemiluminescence reagent (LI-COR Biosciences, Lincoln, NE) was added to the membranes which were imaged with the C-DiGit (LI-COR) blot scanner. Enhanced chemiluminescence reagent (LI-COR Biosciences, Lincoln, NE) was added to the membranes which were imaged with the C-DiGit (LI-COR) blot scanner and the Image Studio (v. 5.0, LI-COR) software.

### Quantification of Contractile Activity and Electrophysiology

Cultures of differentiated cells exhibiting contractile activity were treated with 1 μM isoproterenol or 10 μM forskolin (both from Sigma-Aldrich) for 30 min. Then, the cells were observed under the microscope and beating rates were recorded for at least five foci per condition. Corresponding beating rates before incubation with the aforementioned agents were measured and used to calculate rate changes.

Human stem cell-derived CMs were voltage clamped as described (Bett et al., 2013; Han et al., 2014; Kim et al., 2015; Lin et al., 2015). Currents were recorded using whole-cell clamp at room temperature. Stem cell-derived CM aggregates were attached to 35-mm dishes using 0.1% Matrigel as substrate in 2 ml of XF, and incubated for 3–4 days at 37°C, 5% CO_2_/95% air. Non-adherent aggregates were removed by washing with XF before recording. During recording, cells were continuously perfused with Tyrode solution. Cells were visualized with an Olympus IX73 inverted microscope. Membrane potentials were measured at a temperature of 32°C by whole-cell patch-clamp using an Axon Multiclamp 700B amplifier (Molecular Devices) in current-clamp mode. Patch electrodes were pulled from 1.5 mm diameter borosilicate glass capillaries (Sutter) with a Sutter P-97 microelectrode puller, and had 4–6 MΩ resistances when filled with an intracellular solution that contained 10 mM NaCl, 125 mM KCl, 1 mM MgSO_4_, 5 mM EGTA, 5 mM MgATP, 5 mM Tris-creatine phosphate, 0.3 mM GTP, 10 mM HEPES, with pH adjusted to 7.2 with KOH. Membrane potential data were filtered by the amplifier-incorporated 4-pole Bessel filter at 4 KHz, and digitized at 8 KHz by a Molecular Devices digitizer (Digidata 1440A) using a Dell Optiplex 7020 computer, with pClamp 10 software (Molecular Devices) (Du et al., 2017).

### Statistical Analysis

Data are expressed as mean ± SD unless stated otherwise. ANOVA and *post hoc* Tukey test were performed using Prism (v. 8, GraphPad Software, La Jolla, CA). Values of *p* < 0.05 were considered as significant.

## Results

### Development of Xeno-Free Medium

We set out to develop a simple, xeno-free formulation devoid of factors (e.g., hormones) potentially interfering with cardiogenic differentiation while supporting hPSC growth. An extensive list (Supplementary Table 2) was examined of typical basal media (DMEM, RPMI, DMEM/F12), and components supporting cell proliferation and viability. In formulations with DMEM (Supplementary Figure 1A) different hPSC lines exhibited doubling times that followed the same trend as those for cells maintained in the medium used for routine maintenance of self-renewing hPSCs. Supplementation with recombinant human albumin (rHA) as a protein source (Table 1), resulted in cell viability >85% during 6-day passages. In fact, the proliferation of hPSCs cultured in medium with rHA was similar as in the same base medium containing the more complex free fatty acid mix (FFA; linoleic acid, linolenic acid, and tocopherol acetate), a component of the B27 supplement, or FFA and rHA together (Supplementary Figure 1B).

The rHA-containing formulation, which will be herein referred to as XF, was used for guided differentiation of hPSCs to cardiac cells through biphasic modulation of canonical Wnt signaling (Ueno et al., 2007). Cultures were supplemented with the Wnt activator CHIR99021 (Lian et al., 2012) (henceforth CHIR) inducing mesendoderm specification for 24 h and then with the Wnt inhibitor KY02111 for 6 days. Cells were maintained in XF medium after day 6. By day 13, differentiated H9 hESCs exhibited 23.32 ± 1.4% troponin T-positive (TNNT2^+^) cells followed by 16.17 ± 3.5% and 5.89 ± 5.1%, for IMR90-4 and B12-3 hiPSCs, respectively (Supplementary Figure 1C). Taken together, the XF medium supports cardiogenic specification. However, variability among hPSC lines is pronounced [coefficient of variation (CV) = 57.9%] without optimization.

### Differentiation of hPSCs to Cardiac Mesendoderm in XF Medium

In addition to the observed variability, the low yield in comparison to published reports employing biphasic canonical Wnt signaling modulation was expected given the differences in the base media used and the exclusion of the B27 supplement here. These findings motivated the investigation of select factors known to be involved in driving the commitment of hPSCs to heart cells. This was facilitated by the absence of moieties with cardiogenic potential in the XF medium.

**FIGURE 1:**
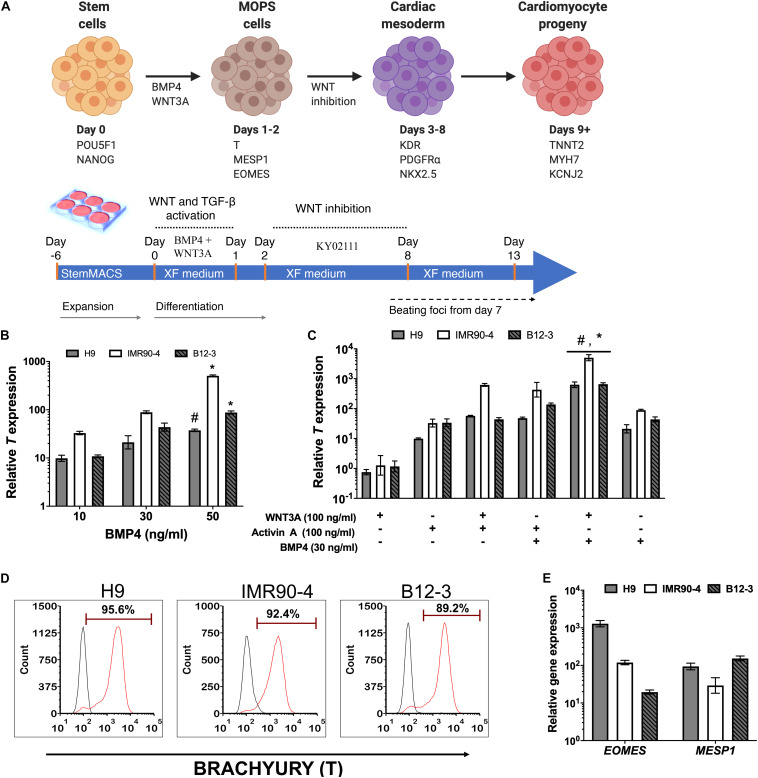
**(A)** Schematic representation of the differentiation stages and process in planar cultures. **(B)** Quantitative PCR (qPCR) for *T* expression at 24 h after treatment of hPSCs with different concentrations of BMP4. #*p* < 0.05, *p < 0.01 vs. treatment with lower BMP4 concentrations for the respective hPSC lines. **(C)** qPCR for *T* expression at 24 h after treatment with different ligand combinations. *p < 0.05 for BMP4 + WNT3A treatment vs. WNT3A only treatment; #*p* < 0.05 for WNT3A + BMP4 treatment vs. BMP4 only treatment (Figure 2B). **(D)** Representative flow cytometry plots for *T* expression by cells derived from the differentiation of H9 (91.1 ± 1.4%), IMR90 (83.51 ± 9.72%), and B12-3 (84.43 ± 10.59%) hPSCs. Black curves: isotype control. Results are mean ± SD from at least three independent experiments. **(E)** Relative expression of early mesoderm genes after BMP4 (30 ng/ml) and Wnt3A (100 ng/ml) treatment of hPSCs for 24 h.

A stagewise approach for *in vitro* differentiation of hPSCs to cardiac cells was considered (Figure 1A) starting with the conversion of hPSCs to mesendodermal cells expressing Brachyury (*T*). Bone morphogenetic protein (BMP) signaling is essential for cardiopoietic lateral plate mesoderm formation (Pereira et al., 2012). To this end, cells exposed to 10–50 ng/ml BMP4 in XF for 24 h consistently exhibited high *T* expression (Figure 1B) while longer treatments did not upregulate *T* further in line with published findings (Zhang et al., 2008). The highest *T* expression was observed at 50 ng/ml BMP4 (Figure 1B) but inclusion of 100 ng/ml WNT3A, which regulates the formation of mesendoderm and mesoderm (Huelsken et al., 2000; Lyashenko et al., 2011), reduced *T* expression in all hPSC lines (Supplementary Figure 2). Addition of WNT3A with 30 ng/ml BMP4 to stem cells for 24 h resulted in higher *T* expression than with WNT3A alone (Figure 1C).

The effect was also explored of activin A, which is present during primitive streak formation (D’Amour et al., 2005; Neilson, 2005) and mesendoderm patterning (Conlon et al., 1994; Ben-Haim et al., 2006; Shen, 2007; Figure 1C). Cells exposed to activin A only or in combination with WNT3A expressed *T* but at lower levels vs. cells treated with BMP4 and WNT3A.

The effectiveness of the BMP4 + WNT3A treatment in XF medium for coaxing hPSCs to mesendoderm was corroborated by flow cytometry (Figure 1D). Guided specification of different hPSC lines displayed low variability (CV = 4.8%) with H9 hESCs yielding 91.1 ± 1.4% (*n* = 3) T^+^ cells while the corresponding fractions for IMR90-4 and B12-3 hiPSCs were 83.51 ± 9.7 and 84.43 ± 10.6%, respectively. Moreover, the resulting transient subpopulation exhibited the mesoderm-oriented primitive streak (MOPS) markers *MESP1* and *EOMES* (Figure 1E). In summary, 30 ng/ml BMP4 and 100 ng/ml WNT3A in XF medium induced the efficient specification of multiple hPSC lines to MOPS cells within 24 h.

### Differentiation of hPSC-Derived MOPS Cells to Cardiomyocyte Progeny

**FIGURE 2:**
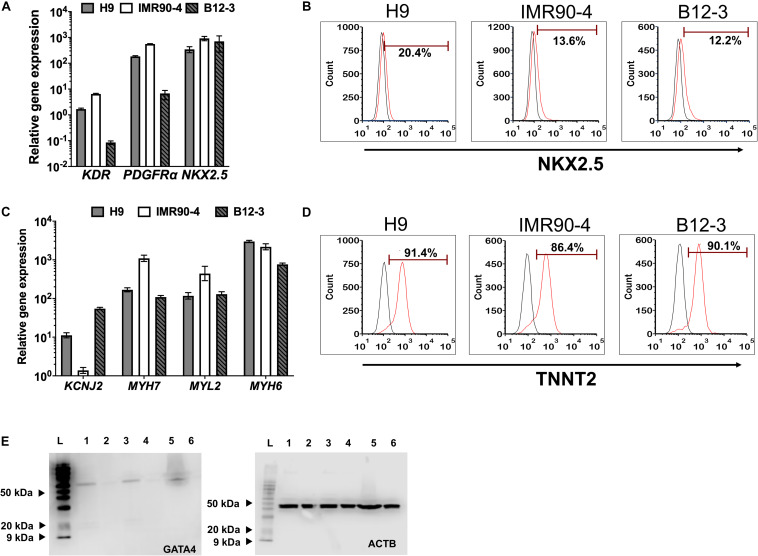
Differentiation of hPSC-derived MoPS cells toward CMs: After 12–24 h of treatment with BMP4 (30 ng/ml) and Wnt3A (100 ng/ml), canonical Wnt signaling is inhibited with KY02111 (10 μM) from days 2–8. **(A)** Expression of early cardiac mesoderm (day 3) in hPSC-derived cells. **(B)** Representative plots of NKX2.5 expression in cells from H9 (21.8 ± 4.08%), IMR90-4 (11.61 ± 2.73%), and B12-3 (7.87 ± 3.8%) cells during transition through the cardiac mesoderm stage (see Figure 1A). Results are shown as mean ± SD (*n* ≥ 3). **(C)** Relative CM gene expression on day 16 of differentiation. **(D)** Flow cytometry analysis of differentiated H9 (91 ± 0.71%), IMR90-4 (81.31 ± 1.32%) and B12-3 (84.57 ± 5.12) hPSCs for TNNT2 expression. Black curve, isotype control. **(E)** Western blot of GATA4 (∼60 kDa) at the end of differentiation for hPSCs (H9:1-2, IMR90-4:3-4, B12-3 hPSCs: 5–6; directed differentiation: 1,3,5; no differentiation factor controls: 2,4,6; L: ladder). ACTB (∼40 kDa) is also shown.

**FIGURE 3:**
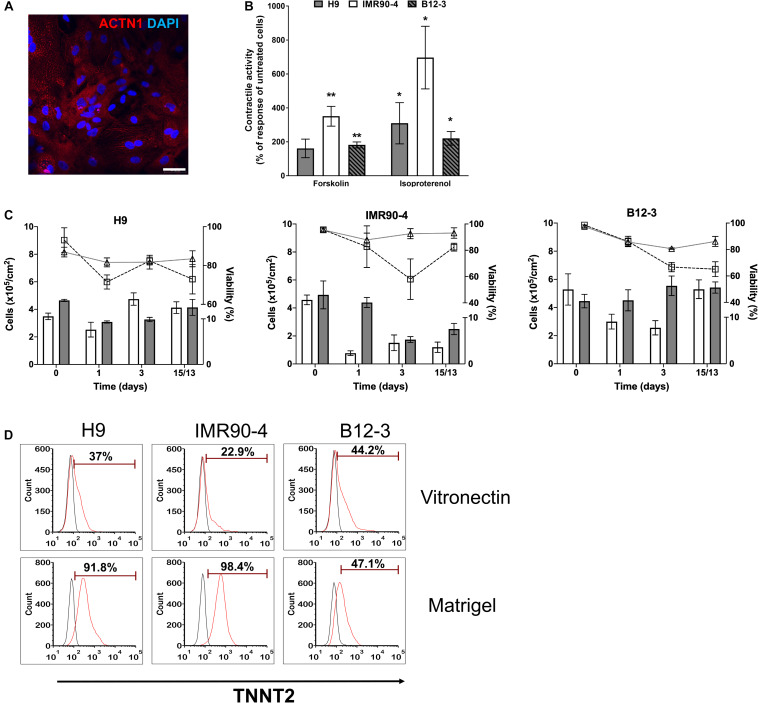
Human PSC-derived CMs in planar culture. **(A)** Immunostaining of differentiated B12-3 cells for ACTN1. Bars: 50 μm. **(B)** Contractile activity of generated CMs in response to forskolin or isoproterenol. *p < 0.05, **p < 0.01 vs. untreated cells. **(C)** Comparison of the number of live cells and viability between the XF protocol developed here (cell number: gray bars; viability: triangles/solid lines) and GW protocol (cell number: white bars; viability: squares/dashed lines) (Lian et al., 2013a). Day 0 denotes the start of the differentiation. **(D)** Flow cytometry data depicting the fractions of TNNT2^+^ cells produced with the GW protocol (day 13) on vitronectin (top row) and Matrigel (bottom row).

Inhibition of canonical Wnt signaling induces cardiac specification of mesoderm regions (Marvin et al., 2001; Schneider and Mercola, 2001) *in vivo* and of cultured hPSC-derived mesoderm cells. To this end, hPSC-derived T^+^ cells were incubated in XF medium with 10 μM KY02111. On day 3 of differentiation, the expression was detected – albeit at different levels among cell lines – of cardiac mesoderm markers *KDR* and *PDGFR*α (Kattman et al., 2011; Figure 2A) concomitantly with the gradual emergence of cells expressing NKX2.5 (Figure 2B). Contractile activity was detected as early as day 7 and became widespread by day 9. The cells expressed markers of CM function including *KCNJ2* (Kir2.1), *MYL2* (MLC2V), *MYH7* [β-MHC), and *MYH6* (α-MHC) (Figure 2C; day 13). By day 13, over 80% of differentiating hPSCs were TNNT2^+^ with comparable efficiencies (Figure 2D; CV = 5.8%). GATA4 expression was evident by western blotting (Figure 2E). Immunocytochemical analysis also confirmed the expression of ACTN1 (α-actinin) after 16 days of differentiation (Figure 3A). Differentiated cells formed foci with contractile activity, which was modulated in an organotypic fashion upon treatment with the β-adrenergic agonist isoproterenol or the adenylyl cyclase activator forskolin (Figure 3B).

The number of cells during various stages of differentiation was comparable between the differentiation with XF medium and that employing a published method (Lian et al., 2013a; Figure 3C). Interestingly, the fractions of TNNT2^+^ cells with the latter method were consistently lower when differentiation was performed on vitronectin instead of Matrigel (Figure 3D) pointing to further optimization for the hPSC lines employed in this study.

Overall, the data show that differentiation of multiple hPSC lines in XF medium with activation of BMP4 and Wnt followed by inhibition of Wnt, results in high fractions of differentiated cells displaying CM markers and beating activity, which can be modulated with chronotropic agents.

### Directed Xeno-Free Differentiation of hPSCs Into Cardiomyocytes in Stirred Suspension

**FIGURE 4:**
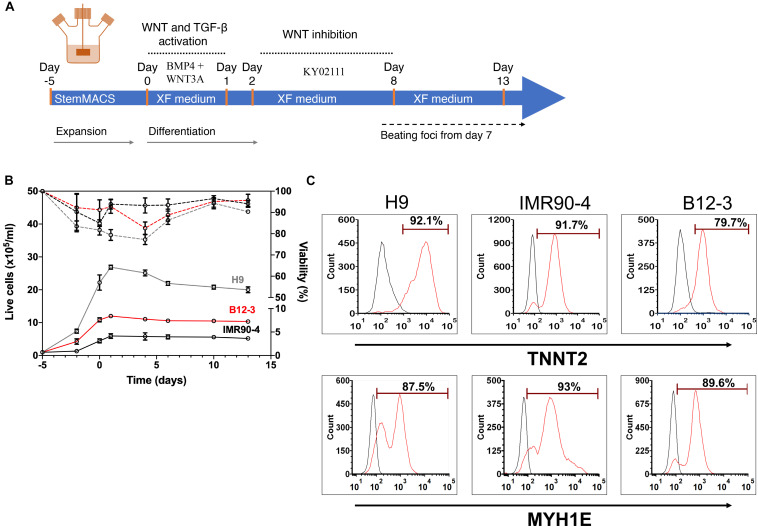
**(A)** Schematic of the hPSC differentiation process in stirred suspension culture. **(B)** Cell proliferation and viability of hPSCs expanded for 5 days and subsequently differentiated toward CMs. Both steps were carried out in spinner flasks. Solid lines represent live cell concentration and dashed lines represent viability. A representative run is shown. H9, gray curves; B12-3, red curves; IMR90-4, black curves. **(C)** Flow cytometry analysis of populations derived from the three hPSC lines on day 13 of differentiation for expression of: TNNT2 – H9: 92.32 ± 1.67%, IMR90-4: 89.13 ± 2.61%, B12-3: 86.69 ± 1.14%, and MYH1E – H9: 84.61 ± 2.59%, IMR90-4: 93.7 ± 3.34%, B12-3: 87.62 ± 3.49% (mean ± SD from *n* ≥ 3). Representative graphs are shown. Sample, red curve; isotype, black curve.

**FIGURE 5:**
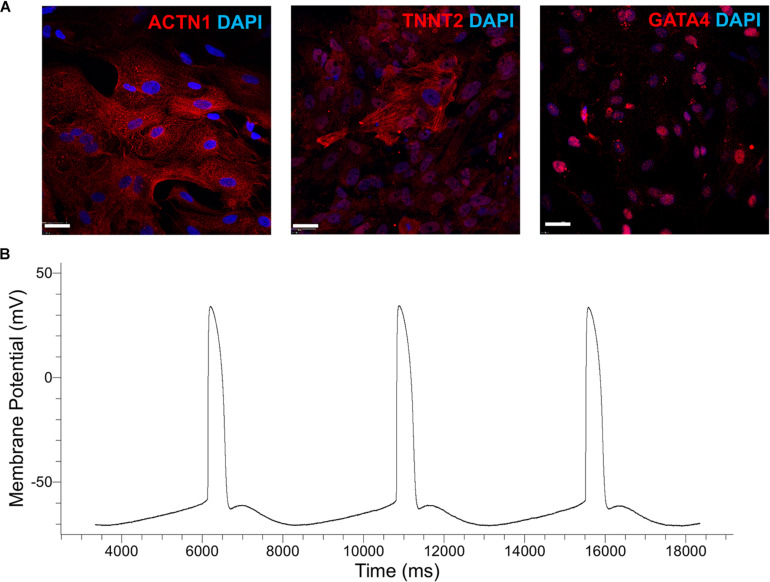
**(A)** Immunostaining of cells generated by the cardiogenic differentiation of hPSCs in spinner flasks. Representative micrographs are shown of ACTN1 (B12-3), TNNT2 (H9), and GATA4 (IMR90-4). DAPI staining of nuclear DNA is also shown. Bars: 50 μm. **(B)** Patch-clamp analysis of H9-derived CMs. A representative action-potential graph is shown.

Next, the XF cardiogenic differentiation of hPSCs developed in static cultures was translated to scalable stirred-suspension cultivation (Figure 4A). First, hPSCs were expanded as aggregates in spinner flasks with medium for regular maintenance for 5 days. Then, differentiation was initiated as described above with XF medium, BMP4 and WNT3A. Cell proliferation decelerated during differentiation resulting in a relatively steady concentration while viability remained >80% (Figure 4B). Interestingly, H9 hESCs continued to increase in number for another day after the start of the differentiation but the subsequent dip brought the concentration to similar level as immediately before the differentiation and this pattern was reproducible (Supplementary Figure 3). After 13 days of differentiation, there were 87–93% TNNT2^+^ cells with a CV of 3.1% among the three hPSC lines (Figure 4C). The corresponding yields were approximately 4.63 (IMR90-4), 10.3 (B12-3), and 20 (H9) TNNT2^+^ cells per seeded hPSC. Similarly, there were 85–94% MYH1E^+^ cells with an interline CV of 5.2% (Figure 4C). The expression of GATA4, ACTN1, and TNNT2 was confirmed by confocal microscopy (Figure 5A).

Patch-clamp analysis was employed to evaluate the functional characteristics of hPSC-derived CMs *in vitro*. Representative images of the recordings show (Figure 5B and Table 2) that the sampled cells had an electrophysiological signature akin to that of ventricular CMs, signaled by the plateau-like phase (Ma et al., 2011). Cells also exhibited a membrane potential and an amplitude similar to those of heart myocytes generated from hPSCs with other protocols (Verkerk et al., 2017). Based on the calculated upstroke, the cells resembled immature CMs, partly because they were tested before day 20 of differentiation (Ben-Ari et al., 2016).

**TABLE 2:** Results of electrophysiological measurements for CMs derived under XF conditions in spinner flask cultures of H9 cells (*n* = 8).

Resting Membrane Potential (mV)	Upstroke (mV/ms)	Peak (mV)	Amplitude (mV)	Max V_*d*_ (mV)	APD50 (ms)	APD90 (ms)
−62.6 ± 4.9	12.2 ± 3.8	48.2 ± 6.3	107.0 ± 5.0	59.8 ± 2.1	351.4 ± 46.8	437.5 ± 97.9

Taken together, these findings demonstrate the successful xenogeneic-free differentiation of hPSCs into cardiac muscle cells in stirred suspension. Notably, the differentiation was characterized by high efficiency for all hPSC lines tested here.

## Discussion

Xeno-free systems are essential for manufacturing therapeutically useful cell types including CMs from hPSCs but the use of complex supplements with fixed or even proprietary composition limits efforts for rapid customization of the differentiation regimen for specific hPSC lines. This is further exacerbated by the largely unexplored role(s), often in a synergistic fashion, of various supplemented moieties on stem cell fate decisions. For instance, trioiodothyronine (T3), which is part of the B27 supplement used in cardiogenic differentiation of hPSCs (Burridge et al., 2011, 2014; Lian et al., 2013a), is also used for the specification of hPSC-derived pancreatic endoderm cells to islet cells (Nair et al., 2019). The components of the XF formulation lack known signaling activities influencing cardiogenic differentiation. This made feasible the detailed assessment of the hPSC induction to CM progeny with factors such activin A, BMP4, and canonical Wnt activators or inhibitors. XF medium with stimuli identified for optimal commitment were used in stirred suspension cultures to generate cells with markers and electrophysiological signature of CMs.

Of note, XF contains insulin that may promote proliferation to partly counter the loss of cells during differentiation. When added early (but not later) in differentiation with activin A and BMP4 and B27 (Lian et al., 2013b), the hormone suppresses the commitment of hESCs to cardiac cell progeny. We also observed lower expression of Brachyury with Activin A and BMP4 vs. BMP4 and WNT3A/CHIR in XF for all three hPSC lines but did not study in detail the potential effect of insulin on hPSC differentiation.

The combined BMP and WNT activation facilitates commitment of hPSCs to mesendoderm in line with the key roles that these pathways play in early cardiopoiesis (Klaus et al., 2007; Parikh et al., 2015). A previous study (Paige et al., 2010) demonstrated that Activin A/BMP4 induction of cardiac mesoderm differentiation is dependent on Wnt signaling. Activin A signals through phosphorylation and activation of Smad2, which is not enhanced by the addition of Wnt3a. However, endogenous Wnt/β-catenin signaling enhances the BMP-mediated production or stability of phospho-Smad1 (Paige et al., 2010) and this may explain the enhanced cardiac specification of hPSCs exposed to BMP4 or combined BMP4/activin. Interestingly, Bmp4 treatment of murine cardiac progenitor cells increases canonical Wnt activity (Andersen et al., 2018). Additionally, BMP signaling induces the transcription of Id1, which in turn mediates cardiac differentiation and the upregulation of relevant genes such as Nkx2.5 and Tbx5 (Katagiri et al., 2002; Cunningham et al., 2017). Better understanding of the interactions among the BMP and canonical WNT pathways will be essential for fine-tuning the differentiation process to compensate for variations in endogenous signaling activity which contribute to the observed discrepancies in the differentiation outcome among hPSC lines (Lian et al., 2012).

Efforts to develop fully defined or xeno-free media for the conversion of hPSCs to cardiac muscle cells have had variable success. An early report on a serum-free defined medium with prostaglandin I_2_ and the p38 MAP kinase inhibitor SB205380 resulted ∼10% of cells in embryoid bodies exhibiting contractile activity and α-MHC (Xu et al., 2008). Through small molecule library screening, Minami et al. (2012) discovered the Wnt inhibitor KY02111, which when supplied in serum-free media, promoted cardiac differentiation of hPSC embryoid bodies in dishes resulting in up to 98% CMs. Yet, there was significant cell loss with only 4.2 × 10^6^ CMs per 6 × 10^6^ hPSCs seeded. This was a similar trend with the XF differentiation of the IMR90-4 cells here. The use of small molecules modulating canonical Wnt activity along with insulin-free B27 was the basis of another report (Lian et al., 2013a) demonstrating the generation of 80–98% cardiac myocytes from hPSCs. In a subsequent improvement (Lian et al., 2015), albumin was eliminated although the concentration and time points of ligand addition varied for different hPSC lines. XF differentiation yielded comparable results with generally improved viability throughout. However, previous methods were developed in static cultures suggesting that further tuning is necessary for adaptation to scalable stirred suspension modalities. Moreover, while the XF formulation involves several components, the overall cost is lower compared to that of other brews for cardiopoietic differentiation, making XF appealing in the context of hPSC bioprocessing.

Direct translation of the cardiogenic differentiation of hPSCs with biphasic modulation of canonical Wnt signaling to stirred suspension vessels has been reported (Kempf et al., 2014; Chen et al., 2015). Differentiation in spinner flask cultures with XF yielded 0.45–2.2 × 10^6^ cells/ml or 0.4–2 × 10^6^ CMs/ml that is comparable to reported yields of 1.5–2 × 10^6^ CMs/ml (Chen et al., 2015) and 0.87 × 10^6^ CMs/ml (Halloin et al., 2019). Moreover, we observed differences among the hPSC lines tested here during their XF specification in stirred suspension. Stem cell proliferation rate depends on multiple factors including genetic background, culture media and conditions, and passage number (Park et al., 2008). For instance, reported doubling times for H9 hESCs vary from around 24 h (Panyutin et al., 2017) to 133 h for a microcarrier culture study (Badenes et al., 2016). Compared to the other hPSC lines, H9 hESCs here exhibiting a greater expansion rate and a decline from a peak concentration of ∼27 × 10^5^ cells/ml a day after commencement of the differentiation to ∼20 × 10^5^ cells/ml at the end. This reduction is lower than a ∼50% loss of cells during a 10-day hPSC differentiation to CMs in automated stirred suspension bioreactors with CDM3 medium (Halloin et al., 2019). Thus, while our study focused on formulating a fully defined, xeno-free medium for the guided cardiopoietic specification of hPSCs, designing respective bioprocesses should entail investigation of the bioreactor-specific culture parameters such as cell density at the onset of differentiation (Kempf et al., 2016), dissolved oxygen (DO) and agitation rate, which impact the potency and purity of derived CMs. Hypoxia appears to improve the cell yield and CM differentiation efficiency of hPSCs cultured as aggregates (Correia et al., 2014) or on microcarriers (Abecasis et al., 2017). Agitation-induced shear influences cell aggregate size in addition to unclear yet mechanotransduction effects on stem cell physiology. Cluster size has been linked to signaling (Chen et al., 2015) and the rate of exchange of nutrients, oxygen, and waste between cells and the medium bulk in bioreactor cultures. Micropatterned aggregate diameter of 400 μm resulted in higher yields of contracting embryoid bodies compared to larger (800 μm) clusters in spinner flasks (Niebruegge et al., 2009). Others reported efficient CM formation upon hPSC expansion in 12-well plates, Erlenmeyer flasks and stirred suspension bioreactors with aggregates measuring on average 210, 400, and 450 μm, respectively (Kempf et al., 2014). Of note, medium in the bioreactor was replenished with a rate of ∼7 ml/2 h. In contrast, the same differentiation failed when hPSCs were expanded with complete exchange of medium every day despite that the resulting aggregates were ∼300 μm. These findings exemplify the need for investigating multiple critical bioprocess parameters including DO regulation, stirring rate, and feeding strategies potentially combined with cell treatment modalities such as encapsulation (Jing et al., 2010; Chang et al., 2020).

It should be noted that the XF differentiation in the bioreactor yielded over 85% CMs without further purification. Previously, lactate, which is utilized effectively by stem cell-derived CMs in culture, was supplemented for their selection over other proliferative cells dependent on glucose for their metabolic needs (Tohyama et al., 2013). Conceivably, a lactate feed late in the differentiation may enhance the overall purity of cardiac cell population. Yet, the impact of cell death and debris on the differentiating cells remains unclear and warrants further investigation.

Overall, we present the development of an open-source XF medium with fully defined composition. The medium is also more economical compared to other reported media even with fewer components (e.g., the formulation in Burridge et al., 2014 with three components is more costly due to its higher concentration of ascorbic acid). The formulation allows customization of guided differentiation depending on the hPSC line under consideration and thus, it constitutes a significant tool for applications involving the use of patient-specific hPSCs. While the XF medium was employed to coax hPSCs toward CMs in scalable stirred suspension cultures, its potential use for manufacturing alternative progenies is an appealing prospect.

## Data Availability Statement

The raw data supporting the conclusions of this article will be made available by the authors, without undue reservation.

## Author Contributions

PA, AP, and ET contributed to study concepts, design, and data analysis. CD carried out to the electrophysiology experiments and collected the respective data. PA and ET wrote the manuscript. All authors contributed to the article and approved the submitted version.

## Conflict of Interest

AP was employed by the company Kite Pharma, a Gilead company. The remaining authors declare that the research was conducted in the absence of any commercial or financial relationships that could be construed as a potential conflict of interest.
