# Supplementary material for: Xenogeneic-Free System for Biomanufacturing of Cardiomyocyte Progeny From Human Pluripotent Stem Cells
Source: Front Bioeng Biotechnol. 2020 Oct 23;8:571425. doi: 10.3389/fbioe.2020.571425 (PMC7644809; doi:10.3389/fbioe.2020.571425)
Supplement: Supplementary file 1 [file Data_Sheet_1.docx]

Supplementary Material

# Supplementary Data

Supplementary Material should be uploaded separately on submission. Please include any supplementary data, figures and/or tables. All supplementary files are deposited to FigShare for permanent storage and receive a DOI.

Supplementary material is not typeset so please ensure that all information is clearly presented, the appropriate caption is included in the file and not in the manuscript, and that the style conforms to the rest of the article. To avoid discrepancies between the published article and the supplementary material, please do not add the title, author list, affiliations or correspondence in the supplementary files.

# Supplementary Figures and Tables

**Suppl. Table 1.** Gene primer sequences (5’ to 3’ orientation)

| Gene | Forward Primer | Reverse Primer | Amplicon  Size (bp) |
| --- | --- | --- | --- |
| *T* | *TGTACTCCTTCCTGCTGGACTT* | *CCCCAACTCTCACTATGTGGAT* | 270 |
| *MESP1* | *CAAGTGACAAGGGACAACTGAC* | *GCTTGCCTCAAAGTGTCTAGC* | 220 |
| *KDR* | *CCAGAAGTAAAAGTAATCCCAGATG* | *CTTTAAAAGTTCTGCTTCCTCACTG* | 246 |
| *PDGFRA* | *ACCTACAAAAACGAGGAAGACAAG* | *CTCTCTCTTGATGAAGGTGGAACT* | 216 |
| *MYH7* | *AGCTAAAGGTCAAGGCCTACAAG* | *AGATCAAGATGTGGCAAAGCTACT* | 213 |
| *MYH6* | GTGGACAAGCTGCAACTGAA | TGTCACTCCTCATCGTGCAT | 215 |
| *MYL2* | *CTCACAATGTTTGGGGAGAAAC* | *CAGGTTCTTGTAGTCCAAGTTGC* | 320 |
| *KCNJ2* | *ACCGCTACAGCATCGTCTCT* | *TCCACACACGTGGTGAAGAT* | 196 |
| *EOMES* | *ACCAAAACACCGATATTACTCAACT* | *TATAATAGCGGGCTTGAGGTAAAGT* | 168 |
| *NKX2.5* | *AGGACCCTAGAGCCGAAAAG* | *AGATCTTGACCTGCGTGGAC* | 243 |
| *ACTB* | *CTTCCTGGGCATGGAGTCCT* | *AGGAGCAATGATCTTGATCTTC* | 202 |

**Suppl. Table 2.** Components tested in the development of the XF formulation.

| Xeno-free Constituent | Concentration | Present in final formulation |
| --- | --- | --- |
| DMEM basal medium (4.5g/L), ThermoFisher Scientific, cat. no. 10569010 | 1x | Yes |
| DMEM basal medium (1.5 g/L), ThermoFisher Scientific, cat. no. 11885076 | 1x | No |
| DMEM/F12 basal medium, ThermoFisher Scientific, cat. no. 12500062 | 1x | No |
| RPMI 1640 basal medium ThermoFisher Scientific, cat. no. 31800022 | 1x | No |
| Human recombinant holo-Transferrin, Millipore Sigma, cat. no. T4132 | 5 µg/ml | Yes |
| Glucose | 15 g/L | No |
| Sodium Selenite, Millipore Sigma, cat. no. S5261 | 5 ng/ml | Yes |
| Recombinant Human Albumin (rHA), Millipore Sigma, cat. no. A9731 | 0.05 – 1 % | Yes |
| Sodium pyruvate, ThermoFisher Scientific, cat. no. 11360070 | 1 mM | Yes |
| MEM Non-essential amino acids, ThermoFisher Scientific, cat. no. 11140050 | 1x | Yes |
| RPMI 1640 Amino acid supplements, Millipore Sigma, cat. no. R7131 | 1x | Yes |
| RPMI 1640 Vitamin supplements, Millipore Sigma, cat. no. R7256 | 1x | Yes |
| Chemically defined Lipid concentrate, ThermoFisher Scientific, cat. no. 11905031 | 1x (1:1000) | No |
| Linolenic acid^(1)^, Millipore Sigma, cat. no. L2376 | 10 ng/ml | No |
| Linoleic acid^(2)^, Millipore Sigma, cat. no. L1012 | 10 ng/ml | No |
| Tocopheryl acetate^(3)^, Millipore Sigma, cat. no. T3376 | 70 ng/ml | No |
| ITS^(4)^, ThermoFisher Scientific, cat. no. 41400045 | 1X | No |
| Insulin^(4)^, Millipore Sigma, cat. no. 91077C | 10 μg/ml | No |
| β-mercaptoethanol^(5)^, ThermoFisher Scientific, cat. no. 21985023 | 10 - 100 μM | No |

Notes:

1. Linolenic acid: Helps prevent cardiomyocyte death (Lanzmann-Petithory, 2001).
2. Linoleic acid: Exhibits activity as an antioxidant (Wey et al., 1993).
3. Tocopheryl acetate: Exhibits activity as an antioxidant (Schafer et al., 2002).
4. Insulin: Induces cell growth and proliferation and aids in glucose metabolism (Straus, 1981), Transferrin: A metal ion transporter, Selenium: required for antioxidant functioning while it promotes cell growth (Zeng, 2009).
5. β-mercaptoethanol: Antioxidant that may promote cell proliferation (Inui et al., 1997).

## Supplementary Figures

**
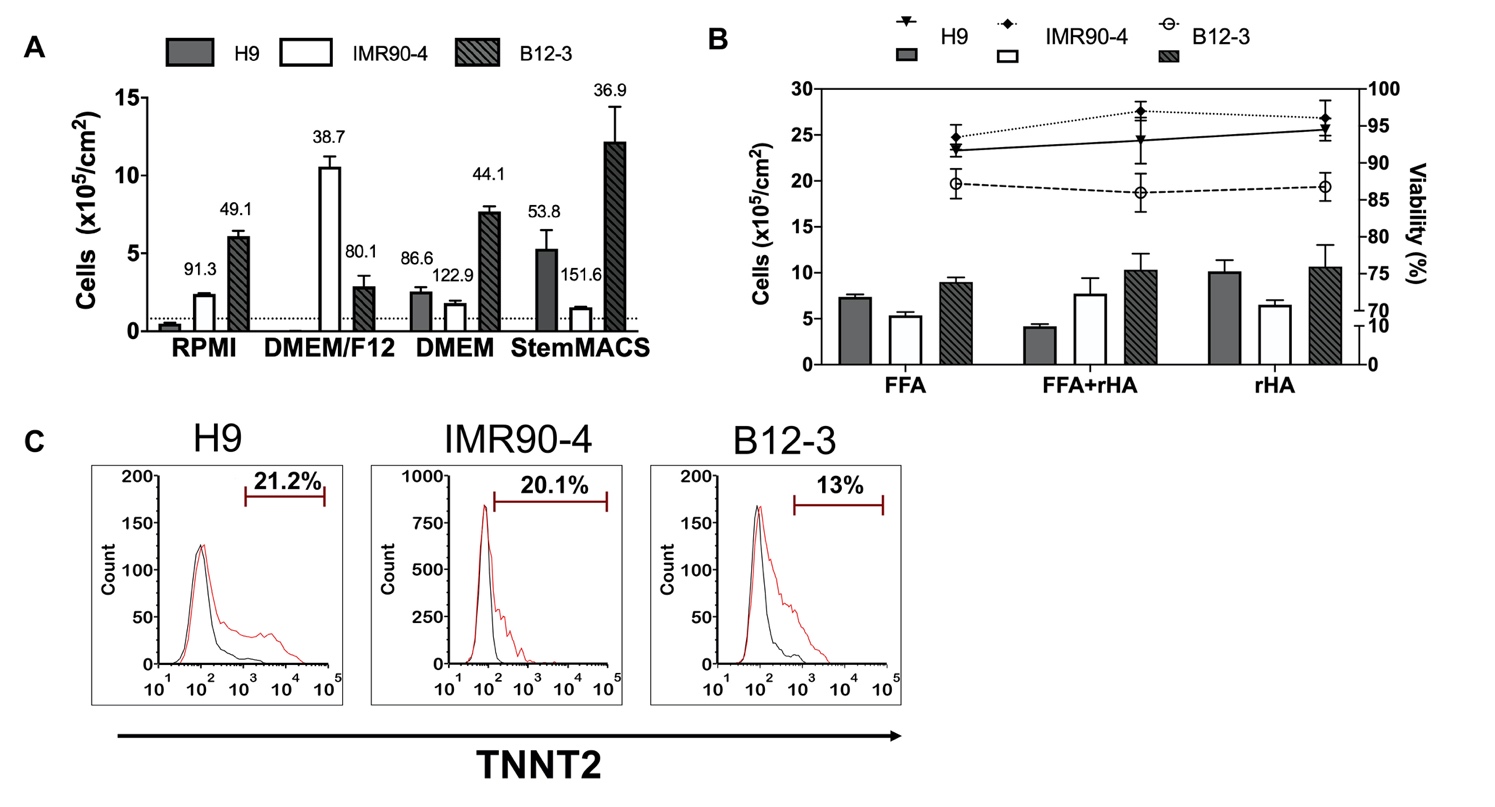
**

**Supplementary Figure S1.** (A) Yields of live H9, IMR90-4, and B12-3 hPSCs in various basal media and medium for routine maintenance (StemMACs). Horizontal dotted line: Seeding density. The numbers above each bar are the apparent doubling times (in h; see Material and Methods). (B) Total cell number (bars) and viability (lines/markers) of hPSCs cultured for 6 days in various differentiation media. (C) Cardiac TNNT2 expression after 13 days of differentiation in rHA-supplemented XF medium with biphasic modulation of Wnt signaling. Representative flow cytometry results (red) with isotype controls (black) are shown.


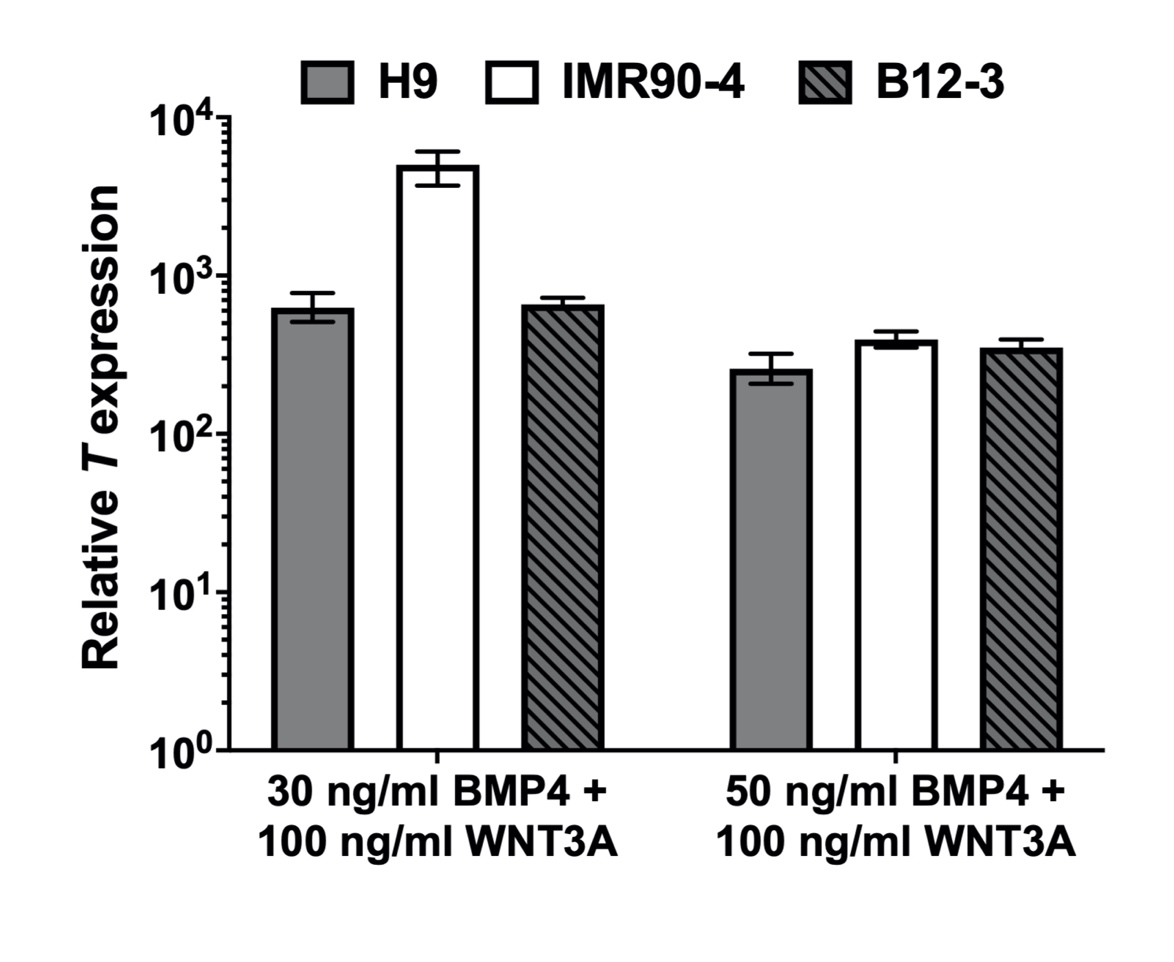


**Supplementary Figure S2.** Relative *T* expression by hPSCs treated with 100 ng/ml WNT3A and 30 or 50 ng/ml BMP4. Results are shown as mean±SD (n=3).


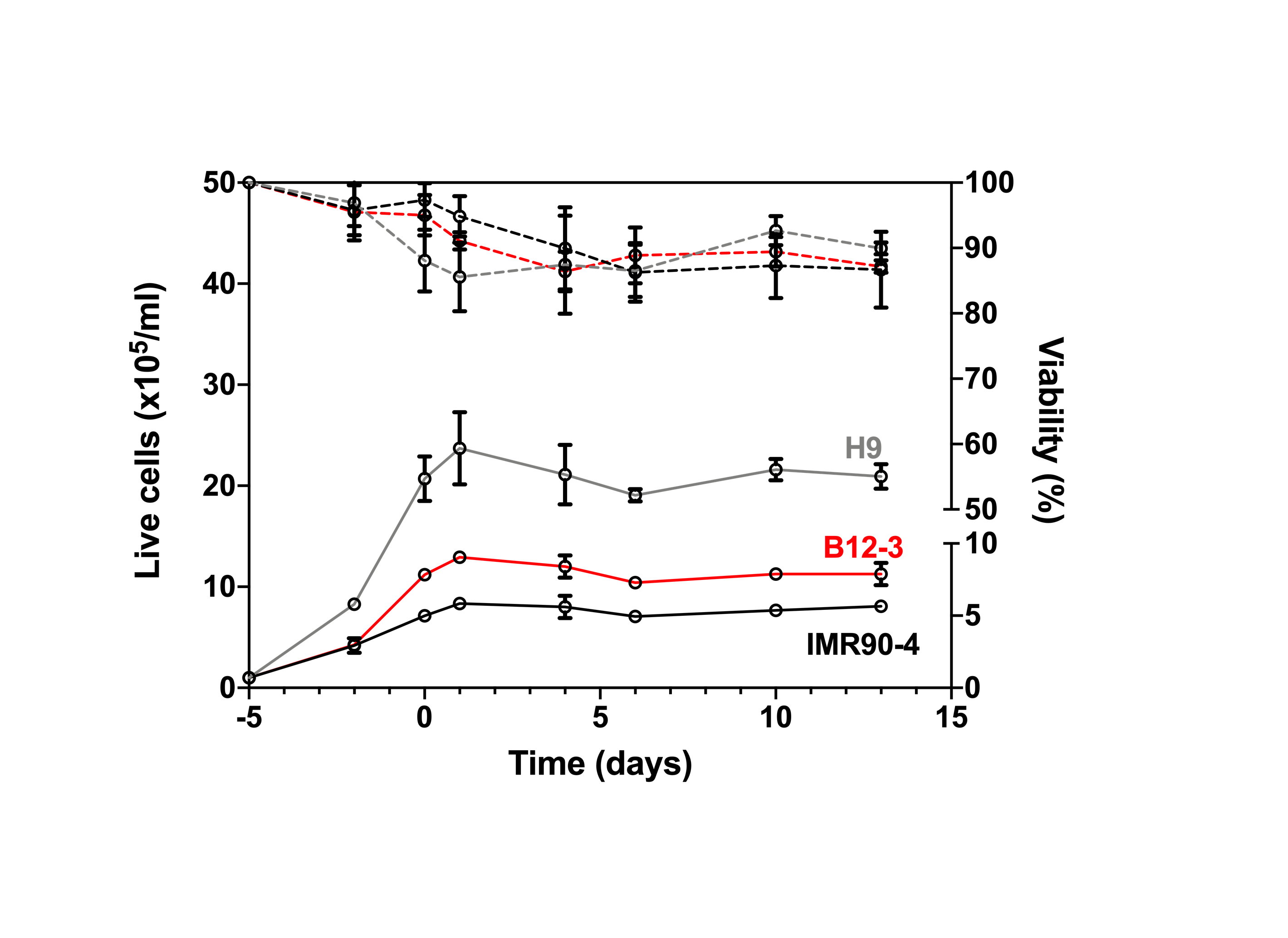


**Supplementary Figure S3.** Representative run of spinner flask expansion and directed differentiation of hPSCs. Results are shown as mean±SD from triplicates.

REFERENCES

Inui, K., Oreffo, R.O., and Triffitt, J.T. (1997). Effects of beta mercaptoethanol on the proliferation and differentiation of human osteoprogenitor cells. *Cell Biol Int* 21(7)**,** 419-425. doi: 10.1006/cbir.1997.0165.

Lanzmann-Petithory, D. (2001). Alpha-linolenic acid and cardiovascular diseases. *J Nutr Health Aging* 5(3)**,** 179-183.

Schafer, F.Q., Wang, H.P., Kelley, E.E., Cueno, K.L., Martin, S.M., and Buettner, G.R. (2002). Comparing β-Carotene, Vitamin E and Nitric Oxide as Membrane Antioxidants. *Biological Chemistry* 383(3-4)**,** 671. doi: <https://doi.org/10.1515/BC.2002.069>.

Straus, D.S. (1981). Effects of insulin on cellular growth and proliferation. *Life Sci* 29(21)**,** 2131-2139. doi: 10.1016/0024-3205(81)90482-3.

Wey, H.E., Pyron, L., and Woolery, M. (1993). Essential fatty acid deficiency in cultured human keratinocytes attenuates toxicity due to lipid peroxidation. *Toxicol Appl Pharmacol* 120(1)**,** 72-79. doi: 10.1006/taap.1993.1088.

Zeng, H. (2009). Selenium as an essential micronutrient: roles in cell cycle and apoptosis. *Molecules (Basel, Switzerland)* 14(3)**,** 1263-1278. doi: 10.3390/molecules14031263.
